# Biomarkers for Predicting Clinical Outcomes of Chemoradiation-Based Bladder Preservation Therapy for Muscle-Invasive Bladder Cancer

**DOI:** 10.3390/ijms19092777

**Published:** 2018-09-15

**Authors:** Fumitaka Koga, Kosuke Takemura, Hiroshi Fukushima

**Affiliations:** Department of Urology, Tokyo Metropolitan Cancer and Infectious diseases Center Komagome Hospital, 3-18-22 Honkomagome, Bunkyo-ku, Tokyo 113-8677, Japan; takemura-uro@cick.jp (K.T.); fukuuro@tmd.ac.jp (H.F.)

**Keywords:** biomarker, chemoradiation, prognosis, bladder preservation, bladder neoplasm, urothelial carcinoma

## Abstract

Chemoradiation-based bladder preservation therapy (BPT) is currently a curative option for non-metastatic muscle-invasive bladder cancer (MIBC) patients at favorable risk or an alternative to radical cystectomy (RC) for those who are unfit for RC. In BPT, only patients who achieve complete response (CR) after chemoradiation have a favorable prognosis and quality of life with a preserved functional bladder. Thus, predicting CR and favorable prognosis is important for optimal patient selection for BPT. We reviewed biomarkers for predicting the clinical outcomes of chemoradiation-based BPT. The biomarkers studied were categorized into those related to apoptosis, cell proliferation, receptor tyrosine kinases, DNA damage response genes, hypoxia, molecular subtype, and others. Among these biomarkers, the Ki-67 labeling index (Ki-67 LI) and meiotic recombination 11 may be used for selecting BPT or RC. Ki-67 LI and erythroblastic leukemia viral oncogene homolog 2 (erbB2) may be used for predicting both the chemoradiation response and the prognosis of patients on BPT. Concurrent use of trastuzumab and a combination of carbogen and nicotinamide can overcome chemoradiation resistance conferred by erbB2 overexpression and tumor hypoxia. Further studies are needed to confirm the practical utility of these biomarkers for progress on biomarker-directed personalized management of MIBC patients.

## 1. Introduction

Bladder cancer is the second most common malignancy of the genitourinary tract after prostate cancer in the United States, with approximately 81,000 new cases and 17,000 deaths each year as of 2018 [1]. Approximately 75% of bladder cancer patients present with non-muscle-invasive bladder cancer confined to the mucosa and submucosa (Tis, Ta, and T1), while the rest present with muscle-invasive bladder cancer (MIBC) [2]. The reference standard of care for MIBC patients has long been radical cystectomy (RC) with urinary diversion and lymph node dissection. However, this surgical procedure is complex and invasive and could have long-term adverse effects on urinary, gastrointestinal, and sexual functions. A recent systematic review on the surgical outcomes of robot-assisted laparoscopic RC demonstrated a 90-day overall complication rate of 59%, a 90-day major complication rate of 15%, and a 90-day mortality rate of 3% [3]. The long-term adverse effects on urinary, gastrointestinal, and sexual functions significantly compromised quality of life (QOL) in patients undergoing RC and urinary diversion when compared with those retaining their native bladders [4].

Bladder preservation therapy (BPT), consisting of transurethral resection, chemotherapy, and radiation, has yielded oncological outcomes and QOL comparable to or more favorable than RC when select MIBC patients undergo BPT [5]. Because of the lack of randomized control trials, BPT used to be an alternative to RC for MIBC patients medically unfit for RC. However, accumulated clinical evidence reported from centers of excellence has demonstrated favorable oncological and QOL outcomes [6,7,8]. Consequently, guidelines of the American Urological Association, American Society of Clinical Oncology, American Society for Radiation Oncology, and Society of Urologic Oncology mention that chemoradiation-based BPT should be offered as an option of standard therapies for non-metastatic MIBC patients who desire to retain their bladders and for whom RC is not a treatment option [2].

MIBC patients with favorable oncological and QOL outcomes after BPT are those achieving complete response (CR) to chemoradiation; such patients account for 50–90% of MIBC patients treated with chemoradiation-based BPT [5]. Although those who do not achieve CR are advised to undergo salvage RC, MIBC patients who do not achieve CR to chemoradiation show unfavorable cancer-specific survival (CSS) regardless of salvage RC with curative intent due to metastatic recurrences [5,9]; such patients may benefit from neoadjuvant chemotherapy plus RC in terms of CSS. Thus, prediction of chemoradiation response enables selection of optimal candidates for BPT and may ultimately improve prognosis and QOL of MIBC patients.

In this study, we conducted a non-systematic review to identify studies investigating biomarkers associated with chemoradiation response and prognosis on chemoradiation-based BPT among MIBC patients. Some biomarkers can be used as targets for therapeutic intervention to improve clinical outcomes by modifying the functions of biomarkers (e.g., concurrent use of trastuzumab for erythroblastic leukemia viral oncogene homolog 2 (erbB2)-overexpressing tumors to improve chemoradiation response).

## 2. Current Practice on Bladder Preservation Therapy in Muscle-Invasive Bladder Cancer Patients

BPT in the form most widely utilized and recommended by guidelines comprises transurethral resection and chemoradiation. Randomized controlled phase III trials demonstrated oncological advantages of concurrent administration of chemotherapeutic agents over radiotherapy alone in non-metastatic MIBC patients. In a Canadian trial reported in 1996, adding cisplatin as a radiosensitizer significantly improved local control [10]. However, this study did not have adequate power to show the survival advantages of chemoradiation over radiotherapy alone. A randomized trial in the United Kingdom investigated fluorouracil and mitomycin-C as radiosensitizers in MIBC patients [11]. The concurrent use of these agents significantly improved the 2-year locoregional disease-free survival from 54% to 67%. In this trial, the 5-year overall survival (OS) improved from 35% to 48% but there was no statistical significance. Case series and phase I/II studies demonstrated possible activity of gemcitabine [12] and paclitaxel [13] as radiosensitizers for MIBC.

### 2.1. Indications

Indications of BPT in MIBC patients are quite different according to whether they are fit for RC or not. BPT for those who are fit for RC and who desire their native bladders is regarded as selective BPT. On the other hand, those who are unfit for RC due to severe comorbidity or poor performance status receive BPT as an alternative to RC.

Selective BPT is generally given to patients with low-risk MIBC in whom favorable oncological outcomes are expected to be comparable to those of RC. Favorable clinical features of MIBC include small, solitary and non-metastatic low stage (clinical T2N0) diseases, the absence of hydronephrosis, and the absence of extensive carcinoma in situ [5]. Such tumors are amenable to complete transurethral resection prior to chemoradiation; indeed, the visibly complete transurethral resection of primary tumors is associated with favorable prognosis in patients treated with BPT [5,8].

In contrast to the RC-fit patients, those unfit for RC would receive BPT with chemoradiation as a curative option alternative to RC. These patients would have diseases of higher risks than those who are subjected to selective BPT. Therefore, RC-unfit patients treated with BPT generally have a worse prognosis than those treated with selective BPT.

### 2.2. Therapeutic Protocols

Two typical templates for chemoradiation were reported: Split- or single-course protocol [5]. A split-course protocol consists of induction chemoradiation of 40–45 Gy, followed by evaluation of any residual tumor with imaging studies and transurethral biopsy, and then consolidative chemoradiation of 20–25 Gy if CR is achieved. Patients who do not achieve CR undergo salvage RC. In contrast, a single-course protocol includes full-dose chemoradiation of 55–65 Gy followed by response evaluation and surveillance if CR is achieved. Although salvage RC is planned for patients who do not achieve CR, high-dose (>60 Gy) pelvic irradiation increases both the morbidity and mortality of RC; in patients undergoing salvage RC after high-dose radiotherapy, mortality rates range from 6% to 33%, higher than the rates reported in published contemporary RC series for non-irradiated subjects, which range up to 4% [5]. The split-course protocol places priority on cancer control by carrying out salvage RC with minimal delay for non-CR patients in selective BPT. A lower dose of preoperative irradiation also reduces the risk of RC-associated complications for non-CR patients after induction chemoradiation [14]. However, splitting of radiotherapy could reduce antitumor effects of the split-course protocol compared with those of the single-course protocol. In this respect, the single-course protocol is more suitable than the split-course protocol for BPT in patients unfit for RC.

Other protocols involve consolidative partial cystectomy with pelvic lymph node dissection following induction chemoradiation [15,16] and intraarterial chemotherapy [17] to increase therapeutic intensity.

### 2.3. Prognosis

A published series of chemoradiation-based BPT reported OS comparable to that of RC (50–70% at 5 years) while preserving the native bladder in 40–60% of MIBC patients [5]. There is no randomized controlled trial comparing outcomes between BPT and RC in MIBC patients. Recently, 3 studies retrospectively compared prognosis of MIBC patients between BPT and RC using a propensity score matching analysis: 2 studies derived from the National Cancer Database (NCD) [18,19] and one study from a Canadian multidisciplinary bladder cancer clinic [20]. The 2 NCD studies demonstrated more favorable OS for RC than that for BPT. Because of the lack of adjustment for performance status, an established prognostic factor in MIBC patients, in these NCD studies, the results would be biased by patients who were unfit for RC due to poor performance status and thus received BPT [18,19]. In the Canadian study, subjects were matched for more prognostic factors including performance status than those of the NCD studies, and BPT yielded OS and CSS similar to RC after propensity score matching [20].

### 2.4. Clinicopathologic Factors Associated with Outcomes of Bladder Preservation Therapy

Clinicopathologic parameters associated with outcomes of chemoradiation-based BPT are classified into tumor and therapeutic factors. The clinical tumor parameters associated with favorable chemoradiation response include small tumor size (<5 cm), clinical T2 stage, unifocal disease, and the absence of hydronephrosis [8,16]. The most important therapeutic parameter is completeness of transurethral resection [8]. Thus, it is mandatory to attempt transurethral resection of tumors as thoroughly as is safely possible for candidates of BPT. Prognostic factors after BPT include completeness of transurethral resection, clinical T stage, and lymphovascular invasion [8].

Coen et al. developed comprehensive nomograms predicting outcomes of BPT including therapeutic response to chemoradiation, CSS, and bladder-intact disease-free survival in a cohort of 325 MIBC patients who were treated with selective BPT of split-course chemoradiation [21]. The most important clinical parameters for CR after chemoradiation are the absence of hydronephrosis and complete transurethral resection followed by younger age (<65 years) and female gender. For CSS, the most favorable parameters include clinical T2 disease (versus T3–4) and the absence of hydronephrosis followed by histological grade 2 disease (versus grade 3). For favorable bladder-intact disease-free survival, the most relevant parameter is the absence of hydronephrosis followed by complete transurethral resection, clinical T2 disease, and younger age (<65 years). Such nomograms may assist patients and clinicians making treatment decisions.

## 3. Biomarkers Associated with Chemoradiation Response and Prognosis

Summaries of studies that reported emerging biomarkers associated with chemoradiation response and prognosis on BPT for MIBC are listed in Table 1 and Table 2, respectively. These biomarkers are categorized as follows: (1) apoptosis-related biomarkers, (2) cell proliferation-related biomarkers, (3) receptor tyrosine kinases (RTK), (4) DNA damage response (DDR)-related biomarkers, (5) hypoxia-related biomarkers, (6) molecular subtypes, and (7) others.

### 3.1. Apoptosis-Related Biomarkers

Apoptosis is a key mechanism by which DNA-damaging stimuli, including chemotherapeutic agents and ionizing radiation, exhibit therapeutic effects. Apoptosis-related biomarkers, including apoptotic index (AI), p53, bcl-2, and bax, were investigated in relation to chemoradiation response and prognosis following BPT. Bax and bcl-2 regulate apoptosis downstream of p53 in response to DNA damage, and bax and bcl-2 have a pro-apoptotic and anti-apoptotic effect, respectively [41]. Rodel et al. reported that a higher AI was associated with a higher CR rate (86% vs. 57%, *p* = 0.02) among 70 invasive bladder cancer (cT1-4) patients treated with chemoradiation (59.4 Gy + cisplatin) [22]. However, no significant association with chemoradiation response was observed for immunohistochemical expression p53 or bcl-2. Neither AI, nor p53, nor bcl-2 was associated with CSS in this study. Matsumoto et al. investigated associations of p53, bcl-2, bax, and apoptotic index with clinical response among 62 invasive bladder cancer patients (cT1G3-T4N0) receiving chemoradiation (median dose 40.5 Gy + cisplatin) [23]. They found no significant association between immunohistochemical expression of each biomarker and chemoradiation response. However, higher bax/bcl-2 ratios were significantly associated with higher CR rates (*p* = 0.029). No association with prognosis was observed for any of these biomarkers.

Thus, some apoptosis-related biomarkers (AI and bax/bcl-2 ratios) are predictive of chemoradiation response but not of prognosis of patients treated with BPT.

### 3.2. Cell Proliferation-Related Biomarkers

Ki-67 is an established marker of cell proliferation and a biomarker reflecting the biological aggressiveness of malignancies. In fact, a high Ki-67 labeling index (LI) was an independent risk factor for recurrence and cancer death in bladder cancer patients undergoing RC [42]. In contrast, Ki-67 LI could be a biomarker predicting favorable clinical outcomes for MIBC patients when they are treated with chemoradiation.

Rodel et al. [22] and Tanabe et al. [24] reported that a higher Ki-67 LI was significantly associated with a higher CR rate among bladder cancer patients receiving chemoradiation. These two studies also demonstrated significantly better CSS for patients with tumors of higher Ki-67 LI. However, Matsumoto et al. reported conflicting results; Ki-67 LI was not associated with chemoradiation response and higher Ki-67 LI was significantly associated with worse CSS [23]. In the latter study [10], the total radiation dose of chemoradiation (median 40.5 Gy) and CR rate (34%) were lower than those of a study by Rodel et al. (59.4 Gy and 71%, respectively) [9]. In a study by Tanabe et al., patients received chemoradiation at 40 Gy, and 73% eventually underwent partial cystectomy of the original MIBC site or salvage RC to completely eradicate possible residual cancer cells [12]. The above conflicting results may be attributed to the difference in therapeutic intensity and the probability of the presence of residual disease; residual cancer cells of high Ki-67 LI, which survive chemoradiation at a relatively low dose, may progress and be associated with worse prognosis in a study by Matsumoto et al. [10].

Magnetic resonance imaging (MRI) is routinely used for staging of bladder cancer. Diffusion-weighted MRI is constructed by quantifying the diffusion of water molecules and malignant lesions exhibit high signal intensity in this modality as a result of higher cellularity, tissue disorganization, and decreased extracellular space, all of which restrict water diffusion [43]. The extent of the water diffusion is quantitatively expressed as apparent diffusion coefficient (ADC) values. Yoshida et al. demonstrated that ADC values were inversely correlated with Ki-67 LI in MIBC tissues and that tumors with lower ADC values were more sensitive to chemoradiation [25]. However, the prognostic significance of ADC values has not yet been assessed among MIBC patients treated with BPT.

Taken together, cell proliferation-related biomarkers (Ki-67 LI and ADC values) are likely to be predictive of the chemoradiation response and prognosis of MIBC patients treated with BPT. However, validation studies are needed to confirm the prognostic significance of Ki-67 LI.

### 3.3. Receptor Tyrosine Kinases

Signaling via receptor tyrosine kinases (RTKs) promotes cell proliferation, tumor invasion, and therapeutic resistance [44]. Among RTKs, erbB2, epidermal growth factor receptor (EGFR), and vascular endothelial growth factor (VEGF)-family proteins are associated with clinical outcomes of chemoradiation in MIBC patients. Notably, a biomarker-driven clinical trial was conducted to overcome chemoradiation resistance of erbB2-overexpressing bladder cancer [28].

Chakravarti, et al. investigated the associations of erbB2 and EGFR with chemoradiation response and prognosis following BPT. They reported that positive erbB2 was significantly associated with lower CR rates (50% vs. 81% for negative erbB2, *p* = 0.026) and that positive EGFR was significantly associated with better CSS (*p* = 0.042) [26]. However, the reasons for the association between positive EGFR and better CSS remain to be elucidated. Inoue et al. confirmed the association between erbB2 overexpression and lower CR rates, and demonstrated, for the first time, adverse CSS for erbB2 overexpression among 119 MIBC patients receiving chemoradiation [27]. These data suggest that erbB2 inhibitors improve clinical outcomes of chemoradiation in patients with MIBC overexpressing erbB2. A recent study by Michaelson et al. showed possible improvement of chemoradiation response with erbB2 targeted therapy [28]; 66 evaluable bladder cancer patients were treated with radiation (64.8 Gy) and either paclitaxel + anti-erbB2 monoclonal antibody, trastuzumab (group 1, *n* = 20) or paclitaxel alone (group 2, *n* = 46) according to the presence (group 1) or absence of erbB2 overexpression (group 2) in tumor tissues. The CR rate at 1 year for group 1 was equivalent to that for group 2 (72% vs. 68%, respectively) with comparable toxicity profiles between the 2 groups. In this phase I/II clinical trial, mid- to long-term prognostic outcomes were not reported.

VEGFs have important functions in tumor angiogenesis and lymphangiogenesis, promoting cancer progression [45]. Two studies demonstrated prognostic significance of expression of VEGF family proteins among bladder cancer patients treated with chemoradiation. Lautenschlaeger et al. reported that overexpression of VEGF-B and C and their receptor VEGFR2 were significantly associated with worse OS [32]. However, no association was observed between these biomarkers and the chemoradiation response. Keck et al. demonstrated that overexpression of VEGF-C or its receptor neutropilin-2 was independently associated with worse OS [33]. In this study, the associations of VEGF-C/neutropilin-2 expression with chemoradiation response were not assessed.

### 3.4. DNA Damage Response-Related Biomarkers

Ionizing radiation induces cell death primarily via DNA double-strand breaks. Upon exposure to ionizing radiation, the damage is detected by the meiotic recombination 11 (MRE11)-RAD50-NBS1 complex, resulting in activation of the DDR pathway [46]. DNA cross-links induced by cisplatin are repaired by the nucleotide excision repair pathway, including excision repair cross-complementing group 1 (ERCC1) [47]. Failure to repair such DNA damage results in tumor cell death.

No DDR-related biomarkers except ERCC1 were associated with chemoradiation response. Kawashima et al. demonstrated that loss of ERCC1 expression was significantly associated with better chemoradiation response (CR rate, 86% vs. 25% for positive ERCC1) in a cohort of 22 MIBC patients [29]. These investigators also showed that loss of ERCC1 expression canceled resistance to ionizing radiation in bladder cancer cells in vitro.

Choudhury et al. reported that higher MRE11 expression was significantly associated with better CSS in 179 invasive bladder cancer patients treated with definitive radiotherapy at 55 Gy (hazard ratio (HR) 0.36, *p* = 0.01) [34]. A trend for an inverse prognostic effect of higher MRE11 expression was observed among patients undergoing RC, and CSS was significantly better for those treated with radiotherapy than RC among patients with tumors of higher MRE11 expression (HR 0.60, *p* = 0.02). This study suggests that MRE11 expression status may allow patient selection for radiotherapy or RC. Sakano et al. reported that positive expression of ERCC1 or X-ray repair cross-complementing group 1 (XRCC1) was significantly associated with better CSS (HR 0.64, *p* = 0.024) among 157 invasive bladder cancer patients undergoing cisplatin-based chemoradiation [35]. Given that chemoradiation sensitivity is a critical surrogate for prognosis among MIBC patients treated with BPT [5,9], findings of this study and the abovementioned ERCC1 study [29] appear to be conflicting. Desai et al. investigated the prognostic impact of DDR gene alterations, which were analyzed using a next-generation sequencing assay, in 48 invasive bladder cancer patients undergoing chemoradiation or radiotherapy [36]. The presence of DDR gene alterations, most commonly identified in *ERCC2*, showed a trend for better bladder or metastatic recurrence-free survival (HR 0.47, *p* = 0.070); this finding is in line with studies on neoadjuvant chemotherapy for MIBC [48,49] and systemic chemotherapy for advanced urothelial carcinoma [50], showing significantly better clinical outcomes for tumors with DDR gene alterations.

Thus, the roles of DNA damage response-related genes or proteins as practical biomarkers for predicting outcomes of chemoradiation seem to be largely unknown. The presence of DDR gene alterations may be a biomarker to predict favorable clinical outcomes of MIBC patients undergoing chemoradiation. MRE11 expression status may be used for selecting BPT or RC. However, further validation studies are needed to confirm the practical utility of DDR-related biomarkers in predicting clinical outcomes of BPT for MIBC patients.

### 3.5. Hypoxia-Related Biomarkers

Tumor hypoxia contributes to tumor progression, invasion, metastasis, and resistance to chemotherapy and radiotherapy [45]. A phase III clinical trial showed that hypoxia modification with carbogen and nicotinamide (CON) significantly improved prognosis of invasive bladder cancer patients treated with radiotherapy [51]. Ad hoc analyses of this clinical trial identified hypoxia-related biomarkers for predicting patients who benefit from concurrent CON [37,38]. Tumor hypoxia induces hypoxia-inducible factor-1α (HIF-1α) expression and necrosis in bladder cancer tissues. The presence of histological necrosis in tumor tissues was predictive of benefit from CON; radiotherapy + CON showed significantly better OS than radiotherapy alone in the presence of necrosis (HR 0.43, *p* = 0.02) but not in the absence of necrosis (HR 1.64, *p* = 0.08) [37]. Similarly, the addition of CON provided survival benefit for patients with higher HIF-1α-expressing tumors (HR 0.48, *p* = 0.02) but not for those with lower HIF-1α-expressing ones (HR 0.81, *p* = 0.5) [38].

### 3.6. Molecular Subtypes

Molecular subtypes of bladder cancer can characterize their clinical behaviors and can help predict therapeutic responses to neoadjuvant chemotherapy prior to RC [52,53]. Tanaka et al. demonstrated that molecular subtypes may be used to predict chemoradiation response in 118 MIBC patients [30]. The subtyping model used was the Lund University model, which characterizes three subtypes based on immunohistochemical expression patterns of cyclin B1 and keratin 5: Urobasal (Uro), genomically unstable (GU), and squamous cell cancer-like (SCC-like) [54]. CR rates after chemoradiation (40 Gy + cisplatin) were 52%, 45%, and 15% for GU, SCC-like, and Uro, respectively (*p* < 0.001). Molecular subtypes were not associated with CSS probably because most non-CR patients underwent salvage cystectomy [30].

### 3.7. Others

Urushibara et al. investigated the associations of heat shock proteins (Hsp), which play key roles in cellular stress responses and can be involved in therapeutic resistance and tumor aggressiveness, with clinical outcomes of 54 invasive bladder cancer patients treated with chemoradiation (40 Gy + cisplatin) [31]. Among Hsp27, Hsp60, Hsp70, and Hsp90, positive Hsp60 expression was independently associated with favorable responses (*p* = 0.05). However, no survival advantage was observed for Hsp60-positive tumors.

Two studies demonstrated the possible utility of serum and blood biomarkers, C-reactive protein (CRP) and lymphocytopenia, in predicting prognosis of MIBC patients treated with BPT [39,40]. Yoshida et al. reported that elevated serum CRP (>0.5 mg/dL) was independently associated with worse CSS (HR 1.8, *p* = 0.046) among 88 MIBC patients undergoing chemoradiation-based BPT [39]. CRP was not associated with chemoradiation response. Joseph et al. reported that lymphocytopenia (<1.5 × 10^9^ /L) was significantly associated with worse disease-free survival (HR 3.9, *p* = 0.003) among 74 MIBC patients receiving chemoradiation (52.5 Gy + gemcitabine) [40].

## 4. Conclusions

**Figure 1:**
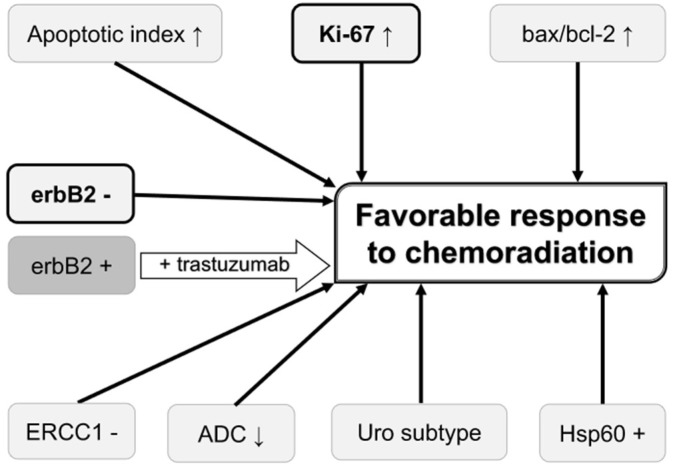
Biomarkers that may be used for predicting and improving the chemoradiation response. Biomarkers in bold letter are also associated with favorable prognosis on bladder preservation therapy. Overexpression of erbB2 is associated with unfavorable chemoradiation response, which can be overcome by trastuzumab. ADC, apparent diffusion coefficient value on diffusion-weighted magnetic resonance imaging; erbB2, erythroblastic leukemia viral oncogene homolog 2; ERCC1, excision repair cross-complementing group 1; Hsp60, heat shock protein 60; Uro subtype, urobasal subtype in the Lund University subtyping model;↑, high value;↓, low value.

**Figure 2:**
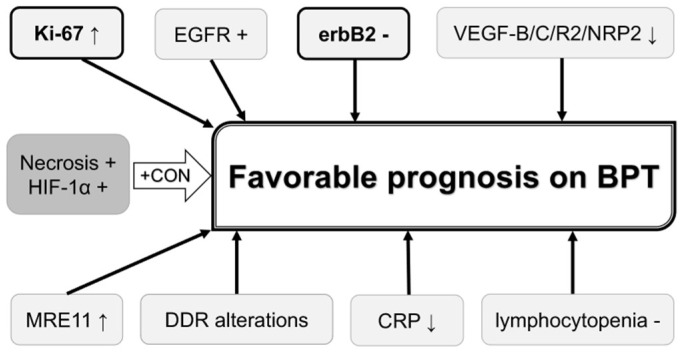
Biomarkers that may be used for predicting and improving prognosis on bladder preservation therapy. Biomarkers in bold letter are also associated with favorable chemoradiation response. Biomarkers in dark shadow are associated with unfavorable clinical outcomes, which can be overcome by CON. BPT, bladder preservation therapy; CON, carbogen and nicotinamide; CRP, C-reactive protein; DDR, DNA damage response; EGFR, epidermal growth factor receptor; erbB2, erythroblastic leukemia viral oncogene homolog 2; HIF-1α, hypoxia-inducible factor-1α; MRE11, meiotic recombination 11; NRP2, neutropilin 2; VEGF, vascular endothelial growth factor;↑, increased expression;↓, decreased expression or low value.

Possible roles of the abovementioned biomarkers in predicting chemoradiation response and prognosis on chemoradiation-based BPT in MIBC patients are summarized in Figure 1 and Figure 2, respectively. Higher Ki-67 LI, negative erbB2, higher expression of MRE11, higher AI, higher Bax/Bcl-2 ratio, lower ADC values, negative ERCC1, Uro subtype in the Lund University subtyping model, positive Hsp60, positive EGFR, lower expression of VEGF-related proteins, presence of DDR gene alterations, lower serum CRP, and absence of lymphocytopenia are possible biomarkers to predict favorable clinical outcomes in these patients. Of these biomarkers, Ki-67 LI and MRE11 may be used for selecting BPT or RC because a higher Ki-67 LI and higher MRE11 expression appear to favor BPT rather than RC in terms of post-therapeutic survival. Ki-67 LI and erbB2 may be used for predicting both chemoradiation response and prognosis of patients on BPT. Concurrent use of trastuzumab and CON can overcome chemoradiation resistance conferred by erbB2 overexpression and tumor hypoxia.

The introduction of immune-oncology drugs (IODs) to daily clinical practices may create a paradigm shift in the management of bladder cancer patients. IODs have the potential to provide long-term survival to a subset of metastatic bladder cancer patients who are considered incurable in the pre-IOD era [55]. Currently, chemoradiation-based BPT is proposed for non-metastatic MIBC patients who desire to retain their bladders and for whom RC is not a treatment option [2]. Hereafter, indications of intensive therapy to the primary site, including chemoradiation and RC, may expand to metastatic MIBC patients for whom long-term survival is expected on IODs. In this respect, identification of biomarkers and development of biomarker panels for comprehensively predicting clinical courses of bladder cancer patients are in increasing demand. In the near future, progress on biomarker research could allow for personalized management of MIBC patients, including those with metastatic disease, eventually providing better prognosis and QOL with BPT and IODs for optimally select patients.

## Figures and Tables

**Table 1:** Biomarkers associated with chemoradiation response.

Biomarkers	Samples Used (No. Patients)	Chemoradiation Regimen	Associations with Response	Study Type	Reference
**Apoptosis-related**					
Apoptotic index	Tumor tissues (*n* = 70)	RT 59.4 Gy + cisplatin	Higher apoptotic index was associated with a higher CR rate (86% vs. 57%, *p* = 0.02)	Retrospective	[22]
bax/bcl-2 ratio	Tumor tissues (*n* = 62)	RT 40.5 Gy (median) + cisplatin	Higher Bax/Bcl-2 ratio was associated with a higher CR rate (*p* = 0.029)	Retrospective	[23]
**Cell proliferation-related**					
Ki-67 LI	Tumor tissues (*n* = 70)	RT 59.4 Gy + cisplatin	Higher Ki-67 LI was associated with a higher CR rate (86% vs. 57%, *p* = 0.02)	Retrospective	[22]
Ki-67 LI	Tumor tissues (*n* = 94)	RT 40 Gy + cisplatin, 69 (73%) underwent partial or salvage radical cystectomy	Higher Ki-67 LI (continuous variable) was associated with a higher CR rate (*p* = 0.0004)	Retrospective	[24]
ADC value	MRI (*n* = 23)	RT 40 Gy + cisplatin	Sensitivity/specificity/accuracy = 92/90/91% when ADC < 0.74 × 10^−3^ mm^2^/s	Retrospective	[25]
**RTKs**					
erbB2	Tumor tissues (*n* = 55)	RT 40 Gy + cisplatin + other agents	CR rates, 50% vs. 81% for positive vs. negative (*p* = 0.026)	Retrospective	[26]
erbB2	Tumor tissues (*n* = 119)	RT 40 Gy + cisplatin	CR rates, 29% vs. 53% for positive vs. negative (*p* = 0.01)	Retrospective	[27]
erbB2	Tumor tissues (*n* = 66)	RT 64.8 Gy + paclitaxel with (group 1: erbB2+) or without trastuzumab (group 2: erbB2-)	CR rates, 72% for group 1 and 68% for group 2	Prospective	[28]
**DDR-related**					
ERCC1	Tumor tissues (*n* = 22)	RT 40-66 Gy + cisplatin or nedaplatin	CR rates, 25% vs. 86% for positive vs. negative (*p* = 0.008)	Retrospective	[29]
**Molecular subtype**					
Molecular subtype	Tumor tissues (*n* = 118)	RT 40 Gy + cisplatin	CR rates, 52%/45%/15% for GU/SCC-like/Uro (*p* < 0.001)	Retrospective	[30]
**Others**					
Hsp60	Tumor tissues (*n* = 54)	RT 40 Gy + cisplatin	Positive Hsp60 was associated with better response (*p* = 0.05)	Retrospective	[31]

RT, radiotherapy; CR, complete response; LI, labeling index; ADC, apparent diffusion coefficient; MRI, magnetic resonance imaging; RTK, receptor tyrosine kinases; erbB2, erythroblastic leukemia viral oncogene homolog 2; DDR, DNA damage response; ERCC1, excision repair cross-complementing group 1; GU, genomically unstable subtype; SCC-like, squamous cell cancer-like subtype; Uro, urobasal subtype; Hsp60, heat shock protein 60.

**Table 2:** Biomarkers associated with prognosis of muscle invasive bladder cancer patients on chemoradiation-based bladder preservation therapy.

Biomarkers	Samples Used (No. Patients)	Chemoradiation Regimen	Associations with Prognosis	Study Type	Reference
**Cell proliferation-related**					
Ki-67LI	Tumor tissues (*n* = 70)	RT 59.4 Gy + cisplatin	Better CSS with preserved bladder for higher Ki-67 LI (50% vs. 36% at 5-year, *p* = 0.04)	Retrospective	[22]
Ki-67 LI	Tumor tissues (*n* = 62)	RT 40.5 Gy (median) + cisplatin	Worse CSS for high Ki-67 LI of > 20% (*p* = 0.014)	Retrospective	[23]
Ki-67 LI	Tumor tissues (*n* = 94)	RT 40 Gy + cisplatin, 69 (73%) underwent partial or salvage radical cystectomy	Better CSS for high Ki-67 LI of > 20% (HR 0.3, *p* = 0.01)	Retrospective	[24]
**RTKs**					
EGFR	Tumor tissues (*n* = 73)	RT 40 Gy + cisplatin + other agents	Better CSS for positive EGFR (*p* = 0.042)	Retrospective	[26]
erbB2	Tumor tissues (*n* = 119)	RT 40 Gy + cisplatin	Worse CSS for erbB2 overexpression (56% vs. 87%, *p* = 0.001)	Retrospective	[27]
VEGF-B/C and VEGFR2	Tumor tissues (*n* = 43)	RT 64.8 Gy + cisplatin + other agents	Worse OS for high VEGF-B/C/R2 expression (*p* = 0.01-0.02), higher distant failure rate for high VEGF-R2 expression (*p* = 0.01)	Retrospective	[32]
VEGF-C/NRP2	Tumor tissues (*n* = 247)	RT 56.3 Gy + cisplatin	Worse OS for high NRP2 or VEGFC expression (HR 4.25, *p* = 0.023)	Retrospective	[33]
**DDR-related**					
MRE11	Tumor tissues (*n* = 179)	RT 55 Gy	Better CSS for high MRE11 expression (HR 0.36, *p* = 0.01)	Retrospective	[34]
ERCC1/XRCC1	Tumor tissues (*n* = 157)	RT 48.6 Gy (median) + cisplatin	Better CSS for positive ERCC1 or XRCC1 (HR 0.64, *p* = 0.024)	Retrospective	[35]
DDR alterations	Tumor tissues (*n* = 48)	RT or chemoradiation (details unavailable)	Trend for better RFS for the presence of DDR alterations (HR 0.37, *p* = 0.07)	Retrospective	[36]
**Hypoxia-related**					
Necrosis	Tumor tissues (*n* = 220)	RT vs. RT + CON	The presence of necrosis predicted better OS for RT + CON than RT alone (HR 0.43, *p* = 0.004)	Retrospective	[37]
HIF-1α	Tumor tissues (*n* = 137)	RT vs. RT + CON	Positive HIF-1α predicted better DFS for RT + CON than RT alone (HR 0.48, *p* = 0.02)	Retrospective	[38]
**Others**					
CRP	Serum (*n* = 88)	RT 40 Gy + cisplatin	Worse CSS for high CRP of > 0.5 mg/dL (HR 1.8, *p* = 0.046)	Retrospective	[39]
Lymphocytopenia	Blood (*n* = 74)	RT 52.5 Gy + gemcitabine	Worse RFS for lymphocytopenia of < 1.5 × 10^9^/L (HR 3.9, *p* = 0.003)	Retrospective	[40]

LI, labeling index; RT, radiotherapy; CSS, cancer-specific survival; HR, hazard ratio; RTK, receptor tyrosine kinases; EGFR, epidermal growth factor receptor; erbB2, erythroblastic leukemia viral oncogene homolog 2; VEGF(R), vascular endothelial growth factor (receptor); OS, overall survival; NRP2, neutropilin 2; DDR, DNA damage response; MRE11, meiotic recombination 11; ERCC1, excision repair cross-complementing group 1; XRCC1, X-ray repair cross-complementing group 1; RFS, recurrence-free survival; CON, carbogen and nicotinamide; HIF-1α, hypoxia-inducible factor-1α; DFS, disease-free survival; CRP, C-reactive protein.
